# An Algorithm to Identify Target-Selective Ligands – A Case Study of 5-HT7/5-HT1A Receptor Selectivity

**DOI:** 10.1371/journal.pone.0156986

**Published:** 2016-06-07

**Authors:** Rafał Kurczab, Vittorio Canale, Paweł Zajdel, Andrzej J. Bojarski

**Affiliations:** 1 Department of Medicinal Chemistry, Institute of Pharmacology Polish Academy of Sciences, 12 Smętna Street, 31–343, Kraków, Poland; 2 Department of Medicinal Chemistry, Jagiellonian University Medical College, 9 Medyczna Street, 30–688, Kraków, Poland; Cincinnati Childrens Hospital Medical Center, UNITED STATES

## Abstract

A computational procedure to search for selective ligands for structurally related protein targets was developed and verified for serotonergic 5-HT_7_/5-HT_1A_ receptor ligands. Starting from a set of compounds with annotated activity at both targets (grouped into four classes according to their activity: selective toward each target, not-selective and not-selective but active) and with an additional set of decoys (prepared using DUD methodology), the SVM (Support Vector Machines) models were constructed using a selective subset as positive examples and four remaining classes as negative training examples. Based on these four component models, the consensus classifier was then constructed using a data fusion approach. The combination of two approaches of data representation (molecular fingerprints vs. structural interaction fingerprints), different training set sizes and selection of the best SVM component models for consensus model generation, were evaluated to determine the optimal settings for the developed algorithm. The results showed that consensus models with molecular fingerprints, a larger training set and the selection of component models based on MCC maximization provided the best predictive performance.

## Introduction

The identification of ligands that display a high affinity for one protein target and that are significantly less active for another or a group of closely related members of a given family is of high relevance for modern drug discovery. Apart from using selective ligands as leads in drug design workflows, they can also be applied as molecular probes for studying, e.g., cellular functions [1]. Because the validation of compound selectivity requires significant experimental efforts and financial resources, fast and accurate computational methods to predict ligand selectivity are highly desirable.

In recent years, diverse computational ligand- and/or structure-based approaches to explain the molecular mechanism of selectivity and/or to predict compound selectivity have been developed. The most prominent example reported on molecular dynamic simulations combined with free energy calculations to study mechanisms underlying the selectivity of tyrosine phosphatases PTP1B/TCPTP/SHP-2 [2], phosphatidylinositol-3-kinases PI3Kα/PI3Kγ [3] and phosphodiesterase PDE5/PDE6 [4]. Other studies have described QSAR modeling to predict the ligand selectivity for serotonin 5-HT_1E_/5-HT_1F_[5] or dopamine D_2_/D_3_ receptors [6] and for a panel of 45 different kinases [7]. Yet other investigations used machine learning (ML) methods to construct selectivity prediction models, e.g., ML based on neural networks to generate structure-selectivity relationship models [8], the binary classification SVM (Support Vector Machines) algorithm to solve multiclass predictions and compound ranking to distinguish between selective, active but non-selective, and inactive compounds [9], and the LiCABEDS (Ligand Classifier of Adaptively Boosting Ensemble Decision Stumps) algorithm to model cannabinoid CB_1_/CB_2_ selectivity [10].

Among fourteen 5-HT receptor (5-HTR) subtypes, 5-HT_7_R represents the most recent addition to a subfamily of G-protein-coupled receptors (GPCRs). Distribution studies revealed a correlation between the localization of 5-HT_7_Rs in the CNS (especially in the suprachiasmatic nucleus) and their function, suggesting that they are involved in the regulation of circadian rhythm, learning and memory processes, as well as in pathological processes such as affective disorders, neurodegenerative diseases, and cognitive decline [11]. A large body of evidence has demonstrated that the clinically established antidepressant effects of atypical antipsychotics, i.e., amisulpiride, lurasidone and aripiprazole, are mediated by antagonism at 5-HT_7_Rs [12,13]. Several preclinical studies support the hypothesis that 5-HT_7_R antagonists may produce beneficial pro-cognitive effects and ameliorate negative symptoms of schizophrenia in animal models [14–17]. On the other hand, potential application for 5-HT_7_R agonists has been proposed for the treatment of dysfunctional memory in age-related decline and Alzheimer’s disease [18], diabetic neuropathy and neuropathic pain [19,20]. Moreover, recent preclinical findings have demonstrated novel therapeutic applications of 5-HT_7_R agonists for the treatment of fragile X syndrome, ADHD and other attention deficit-related diseases [21,22].

Despite a great interest in 5-HT_7_R since the 1990s, its function remains incompletely understood. Apart from fundamental criteria for the classification of receptors, i.e., primary amino acid sequence and signal transduction (G-protein, β-arrestin or MAPK/ERK pathways), 5-HT_7_R displays structural features that are similar to those of 5-HT_1A_R [23–26]. Although this similarity hampers the design of selective ligands of 5-HT_7_R [27,28], the situation appears to be even more complicated when considering the co-localization and functional interplay between 5-HT_7_ and 5-HT_1A_Rs (i.e., homo/hetero dimerization, receptor desensitization and/or internalization) [23,29].

**Fig 1:**
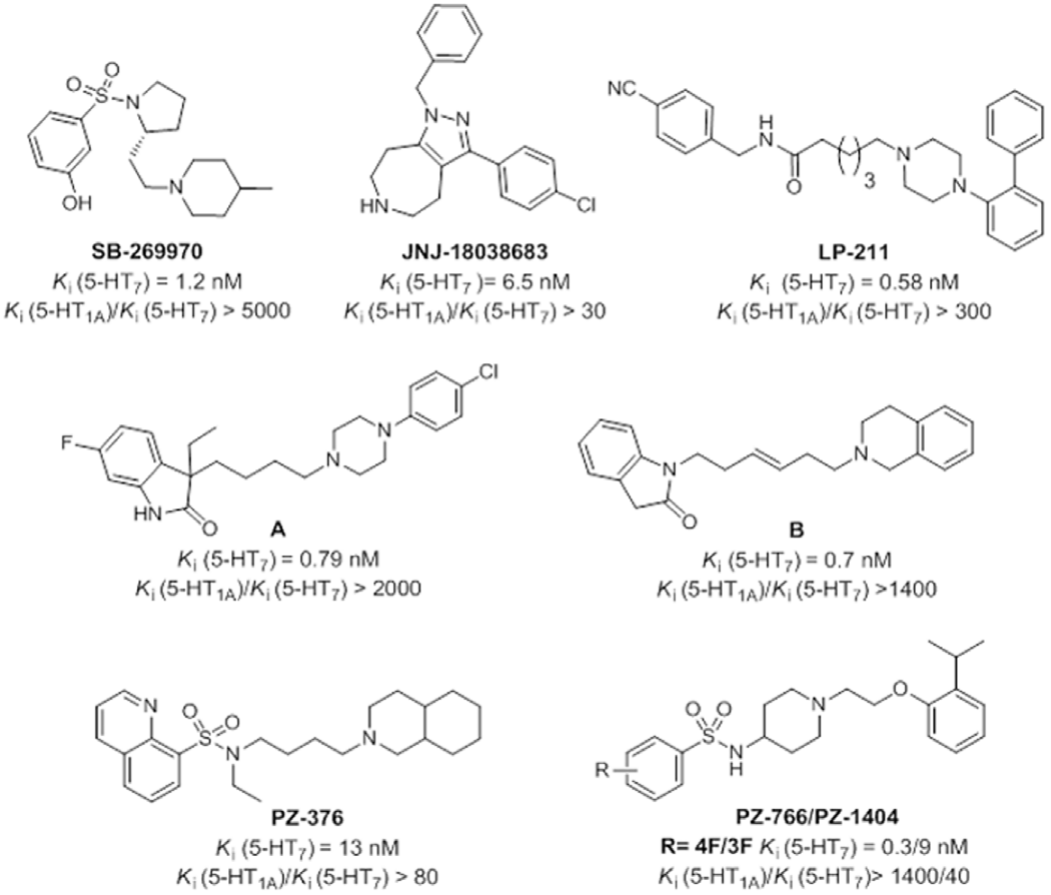
Chemical structure of different chemical classes of selective 5-HT_7_R ligands [30–37].

Considering the aforementioned findings regarding the clinical significance of 5-HT_7_R, the elaboration of new algorithms to support the design of selective 5-HT_7_R agents (as an alternative to those reported in the literature—Fig 1) appears to be critical to obtain a more detailed understanding of the pharmacological function of 5-HT_7_Rs.

**Fig 2:**
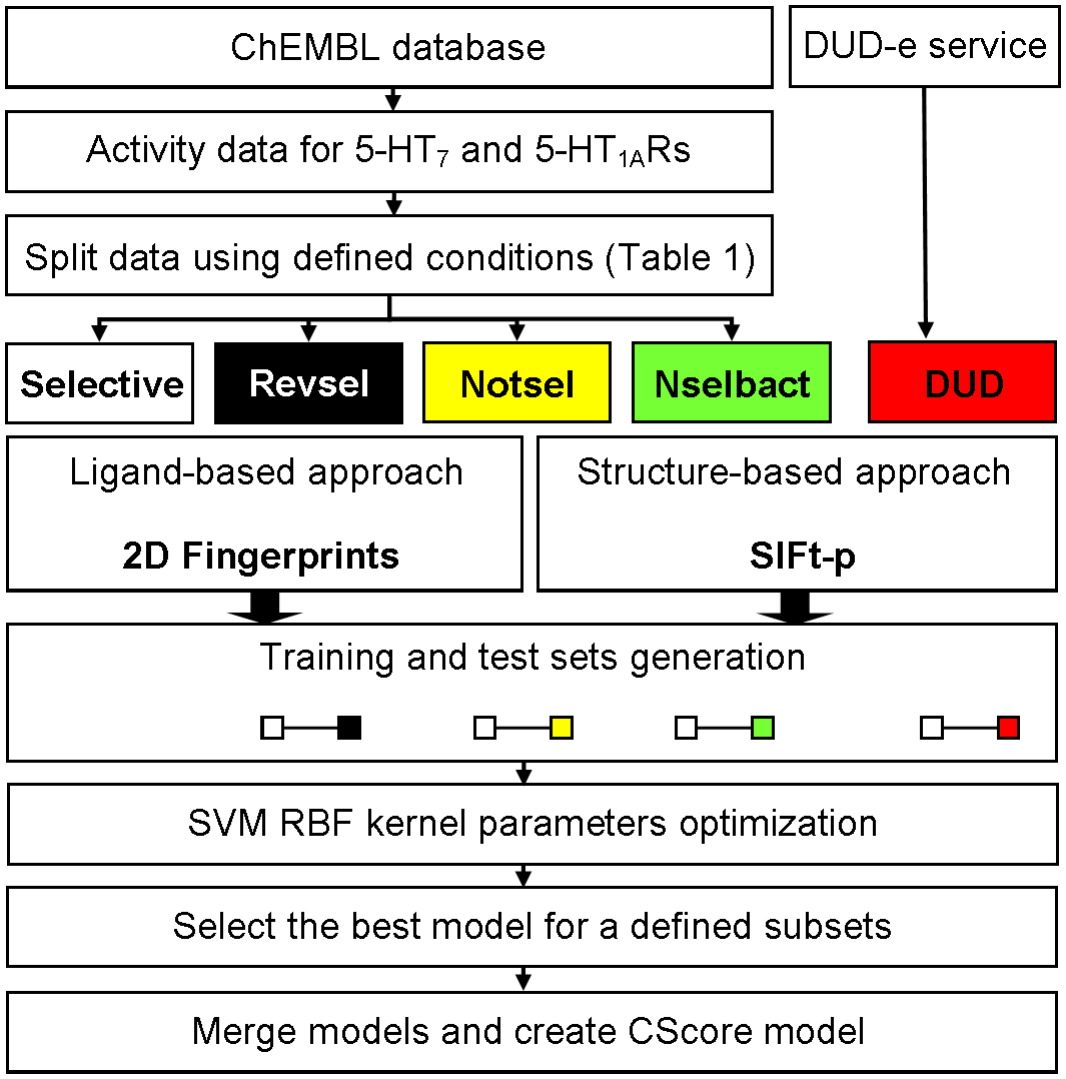
Schematic of the algorithm. The ChEMBL database was filtered out to extract the compounds with annotated activity for both 5-HT_7_ and 5-HT_1A_ receptors. Next, the obtained set of compounds was divided into four subsets using defined rules (Table 1). Additionally, using the DUD-e web service, the decoys for the Selective set were generated. The compounds from each subset were next encoded in binary string format using a set of molecular (ligand-based approach) and interaction fingerprints (SIFt-p, structure-based approach). Next, for each representation, the Selective subset was merged with one from the remaining sets to generate four groups for use in independent training and testing of RBF SVM models (10 trials per issue). The best in-class SVM models were selected based on AUC and MCC values. The final ranking was obtained by application of the SUM rule of data fusion, in which the scores of component SVM models were summed. Abbreviations used: Revsel—reversed selective, i.e., at least 5-fold more active for 5-HT_1A_R than 5-HT_7_; Nselbact—not selective but active, i.e., dual ligands; Notsel—not selective, i.e., the remaining compounds; SIFt-p—Structural Interaction Fingerprints profile (calculated by averaging the SIFts obtained for the docking of a given compound to three target conformations); SVM RBF—Support Vector Machines with radial basis function kernel.

Here, we developed and investigated the algorithm (based on SVM [38] classification models of ligands showing different affinity/selectivity relationships for 5-HT_7_/5-HT_1A_ receptors and a data fusion approach) for its application to predict ligand selectivity between both targets (Fig 2). The performance of this algorithm was compared to a simple ranking strategy and the best in-class component SVM models. Furthermore, ligand- (molecular fingerprints) and structure-based (Structural Interaction Fingerprint, SIFt) data representations, as well as performance metrics (AUC and MCC), were evaluated to select the best SVM models.

## Materials and Methods

### Data sets and definition of training classes

The compounds with activity determined for both 5-HT_1A_ and 5-HT_7_ receptors were retrieved from the ChEMBL v17 database [39]. The parameters (*K*_i_, IC_50_ and p*K*_i_) describing the ligand affinity were used to define four subsets of compounds (Table 1), i.e., selective toward 5-HT_7_R (Selective) or 5-HT_1A_R (Revsel), not-selective but active (Nselbact) and not-selective (Notsel). The list of compound ChEMBL IDs belonging to a given subset is provided in the Supporting Information (S2 File).

**Table 1:** Definitions of SVM training sets used for component model generation.

Set Name	Description/Conditions used to generate the set	Size	SVM class	Inter-class Tc similarity^a^
**Selective**	Selective ligands for 5-HT_7_R:*K*_i_(5-HT_7_) < 100 nM & *K*_i_(5-HT_1A_)/*K*_i_(5-HT_7_) > 5	69	Positive	0.33
**DUD**	Decoy set generated for selective set using DUD-e service	5198	Negative	0.25
**Revsel**	Reverse selective ligands (selective ligands for 5-HT_1A_R):*K*_i_(5-HT_1A_) < 100 nM & *K*_i_(5-HT_7_)/*K*_i_(5-HT_1A_) > 5	124	Negative	0.29
**Nselbact**	Active ligands for both receptors simultaneously:*K*_i_(5-HT_7_) & *K*_i_(5-HT_1A_) < 100 nM	89	Negative	0.31
**Notsel**	Ligands that do not meet the condition of selectivity:0.20 < *K*_i_(5-HT_1A_)/*K*_i_(5-HT_7_) < 5	440	Negative	0.29

^a^ An average Tanimoto coefficient obtained by pairwise comparison (compounds encoded by CDK fingerprint) for each molecule of the Selective subset with each molecule of the remaining subsets. All average pairwise intra- and inter-class Tc are presented as a heat map in S1 Fig.

The p*K*_i_ and IC_50_ values were recalculated to the *K*_i_ using the following expressions: *K*_i_ = 10^–p*K*i^ and *K*_i_ = IC_50_/2. The conversion factor of 2 was suggested by Kalliokoski et al. [40] and has been applied successfully in similar studies [41,42]. In addition, for each selective ligand, 50 decoys with similar 1D physicochemical properties to remove bias (e.g., molecular weight, logP) and a dissimilar 2D topology to be likely non-binders, were generated using DUD-e service [43]. Accordingly defined sets were further used to construct component (class-specific) SVM models by combining the Selective subset (positive learning examples) with DUD, Revsel, Notsel and Nselbact (negative learning examples).

### Data representation

Two approaches, i.e., ligand-based and structure-based approaches were tested to identify the optimal way for constructing selectivity prediction models. In the ligand-based approach, the structure of a molecule was encoded by three different molecular fingerprints (FP): hashed FP [44] (CDK FP, 1024 bits), Klekota-Roth FP [45] (KlekFP, 4860 bits) and MACCS FP [46] (MACCSFP, 166 bits), which were calculated using PaDEL-Descriptor software [47].

**Fig 3:**
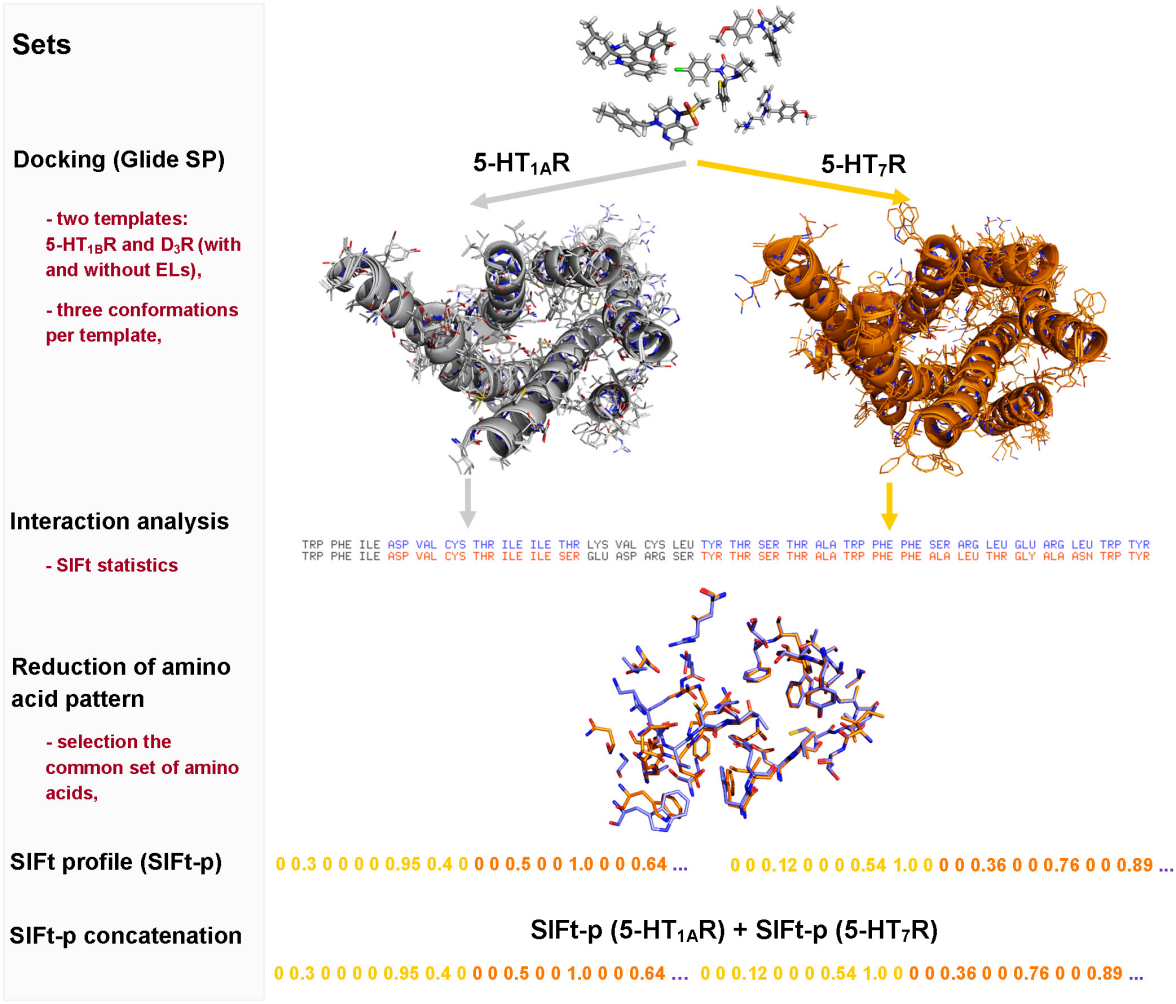
Schematic of the structure-based approach. In the first stage, all subsets of compounds were docked (Glide SP mode) to the three conformations of the 5-HT_1A_R and 5-HT_7_R homology models (generated on 5-HT_1B_R and D_3_R templates). Next, the interaction fingerprints (SIFt) were calculated for all obtained ligand-receptor complexes. The interaction analysis resulted in the selection of 34 common amino acids that formed any type of interaction with the ligands. For a given compound, the SIFts were recalculated (independently for each receptor and template) and averaged (SIFt-p). Finally, for each compound, the reduced SIFt profile (concatenating dockings to 5-HT_1A_ and 5-HT_7_ receptors to a single vector) was used as an input to SVM.

In the structure-based approach (Fig 3), Structural Interaction Fingerprint profiles (SIFt-p) were used [48,49]. They were obtained by docking all the defined subsets of ligands to different conformations of 5-HT_1A_ and 5-HT_7_Rs homology models [26,50] with and without extracellular loops (EL). The 3-dimensional structures of the ligands were prepared using LigPrep v3.6 [51], and the appropriate ionization states at pH = 7.4 were assigned using Epik v3.4 [52]. The Protein Preparation Wizard was used to assign the bond orders, appropriate amino acid ionization states and to check for steric clashes. The receptor grid was generated (OPLS_2005 force field) by centering the grid box with a size of 15 Å on Asp3.32. Automated docking was performed using Glide v6.9 at the SP level with the flexible docking option turned on [53]. Five poses per ligand were generated, but only the one with the best Glide Score was used for SIFt generation.

The SIFt encodes the 3D ligand-protein complex in the form of a 1D binary string, in which a nine-bit pattern is used to describe the interaction type: any contact, backbone, side chain, polar, aromatic, hydrophobic interaction, hydrogen bond donor/acceptor and charged [54]. The SIFt-p were created by calculating the mean value for each position in three fingerprint strings obtained for a given compound in three conformations of a given receptor model. Based on the docking of all compounds and sequence alignment of 5-HT_1A_ and 5-HT_7_ receptors, a set of 34 common amino acids (sharing the same sequence position and having any contact with the docked set of ligands) was defined, and the reduced SIFt-p were created. Finally, for each compound, the reduced SIFt profiles from docking to 5-HT_1A_ and 5-HT_7_ receptors were concatenated to a single vector that handles information regarding averaged interactions between a particular ligand and both receptors.

### Optimization of SVM learning parameters

**Fig 4:**
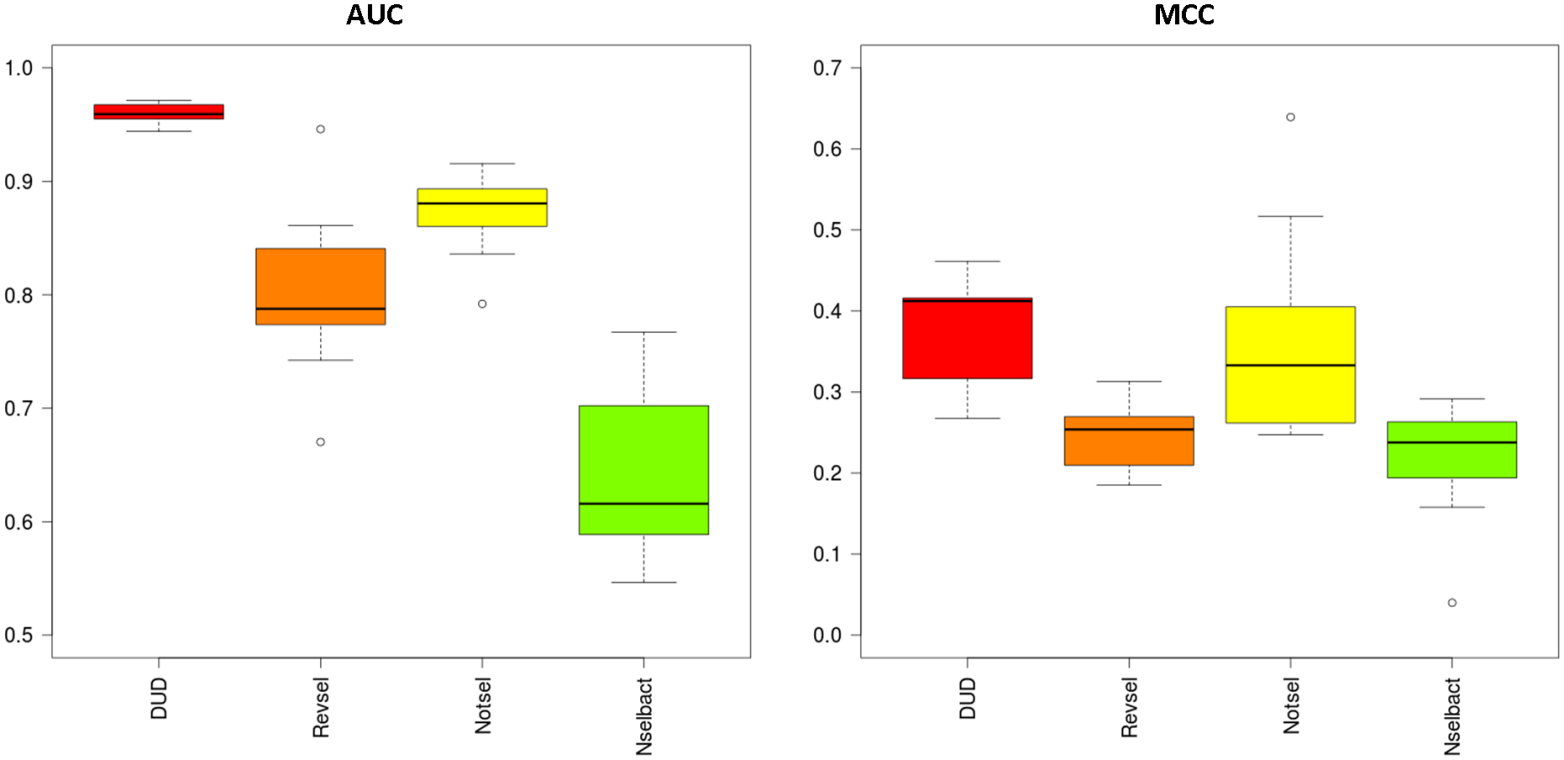
Box plots illustrating differences in performance (AUC and MCC) of 10 optimized SVM component models built for all training classes generated for MACCS FP and a training ratio of 0.40.

The molecular fingerprints and concatenated SIFt-p were used as input to generate classification models using the SVM algorithm. To select the classification model with the best performance for a given training class, a bootstrapping procedure was used. All classes were divided into training and test sets using two ratios: 0.40 and 0.60 (i.e., 40% and 60% of all examples, respectively, were used for training, while the remaining ones constituted the testing set). For each ratio, 10-trials of resampling with replacement of the original sets was performed to optimize the kernel parameters in the SVM classification model. The models were constructed using the SVMlight library and radial base function (RBF, the Gaussian function) [55]. For each run, a grid search was performed for two parameters: a penalty of the error term C ∈ {10^−4^, 10^−3^, 10^−2^, 0.5, 0.1, 1, 5, 10, 100, 500, 1000} and gamma coefficient for the radial base function γ ∈ {10^−7^, 10^−6^, 10^−5^, 10^−4^, 10^−3^, 10^−2^, 10^−1^, 0, 1, 5, 10, 100}. The example distribution of AUC and MCC values for the 10 best SVM models optimized for a training ratio of 0.40 and MACCS FP is presented in Fig 4.

### CScore model generation

**Fig 5:**
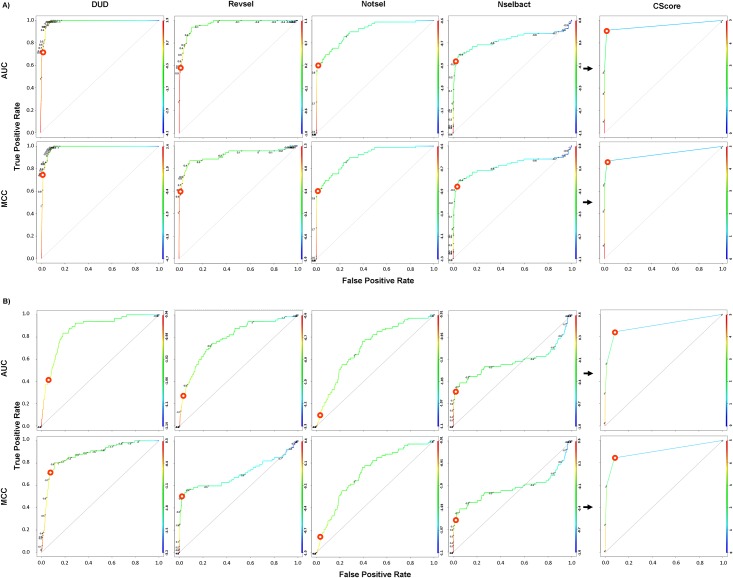
Example illustration of CScore model generation using component models. The ROC curves were used to show the performance of the component and CScore models and the thresholds (red circles) used to determine the classification (A—for MACCS FP and a training ratio of 0.40; B—for SIFt-p generated using the 5-HT_1B_R template with loops and a training ratio of 0.40).

For each set of optimized SVM component models, the threshold (boundary value that separates positive and negative classes) for which MCC was highest was determined by sampling the range of the RBF decision function with a step of 0.1. Next, the best SVM models were identified and used to build the consensus score (CScore) classifiers (Fig 5). Two criteria were used to select the best in-class SVM model—the highest AUC or MCC values. The set of best component models and their thresholds were then used to perform the new classification. The CScore model was created by applying the SUM rule from the data fusion [56] merging the outputs of the individual component classifiers by summing the predictions that were calculated using the thresholds obtained for each component model.

### The performance measures

The recall (1), precision (2), Mathews Correlation Coefficient—MCC (3), area under the receiver operator characteristic (ROC) curve (AUC) and the Boltzmann-Enhanced Discrimination of ROC (BEDROC) metrics were used to assess the classification effectiveness of trained SVM models.

$$R=\frac{TP}{TP+FN}$$ (1)

$$P=\frac{TP}{TP+FP}$$ (2)

$$MCC=\frac{TP\cdot TN-FP\cdot FN}{\sqrt{\left(TP+FP)\cdot (TP+FN)\cdot (TN+FP)\cdot (TN+FN\right)}}$$ (3)

BEDROC=∑i=1ne−αriNnN(1−e−α)(eαN−1)×nNsinh(α2)cosh(α2)−cosh(α3−αnN)+11−e−α(1−nN) (4)

where *TP* denotes the number of true positives (actives labeled as actives), *TN*–true negatives, *FP*–false positives (inactives labeled as actives), *FN*–false negatives, *n* is the number of actives among N compounds, *ri* is the rank of the *i*-th active and α is a parameter that assigns a weight towards compounds the top of the ranked list.

Recall measures the number of correctly identified positive examples, precision describes the correctness of positive predictions and MCC is a balanced measure of the binary classification effectiveness, ranging from –1 to 1, with 1 indicating a perfect prediction. The ROC presents the variation in the number of correctly classified positive examples with the number of incorrectly predicted negative examples. The BEDROC was introduced by Truchon and Bayly [57] to address the problem of "early recognition" in virtual screening. It can be interpreted as the probability that an active is ranked before a randomly selected compound that is exponentially distributed with parameter α, which controls the earliness of "early recognition" to test whether a ranking method is useful in the context of VS. The BEDROC metric ranges from 0 to 1, and it was calculated for α = 20 in the present study, as previously suggested [58].

The ROC curves and AUC values were calculated using the ROCR [59] package in R [60]. The BEDROC was also calculated in R using the enrichvs package [61].

## Results

It should be emphasized that in the present analysis we focused on comparing the performance of the designed algorithm in different settings (i.e., representations, selection of the best component models) in terms of its ability to distinguish Selective from not-selective, inactive, decoy and multimodal (dual) compounds for the virtual screening of molecular databases. Moreover, the classification obtained by combining the SVM and binary molecular fingerprints cannot be interpreted at the level of chemical features that may be responsible for compound selectivity.

Initially, the performance of the CScore, the best component and Classical (trained on the Selective subset as positive class and on the sum of the Revsel, Notsel and Nselbact subsets as negative classes) were compared. The AUC, BEDROC and MCC parameters were calculated (Table 2) for ligand-based and structure-based approaches.

**Table 2:** Performance of CScore models obtained for the 0.40 training ratio and AUC and MCC strategies compared with the best single models trained in the classical manner and the best in-class component models. The median values for the Classical and best component strategies (ten trials were performed) are presented in parentheses.

Fingerprint	Strategy	BEDROC	MCC	AUC
CDK FP				
MCC	Best Classical	0.82 (0.72)	0.60 (0.55)	0.97 (0.92)
	Best component (notsel)	0.77 (0.74)	0.64 (0.53)	0.94 (0.91)
	CScore	0.93	0.65	0.93
AUC	Best Classical	0.84 (0.73)	0.53 (0.52)	0.96 (0.91)
	Best component (dud)	0.72 (0.68)	0.48 (0.55)	0.95 (0.94)
	CScore	0.92	0.52	0.96
MACCS FP				
MCC	Best Classical	0.81 (0.76)	0.66 (0.61)	0.93 (0.91)
	Best component (notsel)	0.77 (0.70)	0.64 (0.50)	0.90 (0.92)
	CScore	0.95	0.72	0.94
AUC	Best Classical	0.76 (0.75)	0.68 (0.66)	0.92 (0.91)
	Best component (dud)	0.82 (0.78)	0.66 (0.57)	0.98 (0.97)
	CScore	0.93	0.74	0.95
KlekR FP				
MCC	Best Classical	0.78 (0.76)	0.69 (0.61)	0.90 (0.89)
	Best component (dud)	0.77 (0.75)	0.72 (0.60)	0.93 (0.93)
	CScore	0.91	0.69	0.88
AUC	Best Classical	0.82 (0.81)	0.60 (0.61)	0.95 (0.94)
	Best component (dud)	0.86 (0.80)	0.63 (0.60)	0.97 (0.96)
	CScore	0.90	0.69	0.96
SIFt-p_D_3__loop				
MCC	Best Classical	0.12 (0.11)	0.17 (0.05)	0.61 (0.55)
	Best component (notsel)	0.45 (0.13)	0.33 (0.17)	0.60 (0.59)
	CScore	0.90	0.50	0.92
AUC	Best Classical	0.12 (0.11)	0.07 (0.05)	0.68 (0.63)
	Best component (dud)	0.24 (0.22)	0.15 (0.13)	0.71 (0.68)
	CScore	0.67	0.13	0.77
SIFt-p_D_3__nloop				
MCC	Best Classical	0.16 (0.13)	0.09 (0.05)	0.61 (0.55)
	Best component (nselbact)	0.41 (0.35)	0.43 (0.25)	0.46 (0.42)
	CScore	0.71	0.27	0.81
AUC	Best Classical	0.15 (0.13)	0.09 (0.05)	0.65 (0.60)
	Best component (revsel)	0.40 (0.39)	0.25 (0.21)	0.71 (0.64)
	CScore	0.53	0.11	0.75
SIFt-p_5-HT_1B__loop				
MCC	Best Classical	0.65 (0.22)	0.59 (0.13)	0.85 (0.77)
	Best component (revsel)	0.56 (0.16)	0.39 (0.11)	0.82 (0.68)
	CScore	0.81	0.50	0.90
AUC	Best Classical	0.63 (0.22)	0.65 (0.11)	0.82 (0.76)
	Best component (dud)	0.39 (0.37)	0.20 (0.22)	0.83 (0.80)
	CScore	0.75	0.53	0.90
SIFt-p_5-HT_1B__nloop				
MCC	Best Classical	0.12 (0.14)	0.12 (0.06)	0.65 (0.65)
	Best component (revsel)	0.49 (0.44)	0.37 (0.34)	0.51 (0.50)
	CScore	0.72	0.39	0.85
AUC	Best Classical	0.23 (0.14)	0.12 (0.06)	0.69 (0.61)
	Best component (dud)	0.40 (0.37)	0.17 (0.11)	0.81 (0.77)
	CScore	0.67	0.24	0.82

Interestingly, to address the “early recognition” problem (BEDROC value), the CScore models always demonstrated superior performance for recognizing selective over not-selective compounds in comparison to any single SVM-based ranking strategy (i.e., Classical and best component). However, considering the global performance (MCC, AUC), a single strategy sometimes provided better results than a consensus approach. In should be noted that use of MCC as the SVM model selection strategy consistently provided better CScores than AUC.

**Fig 6:**
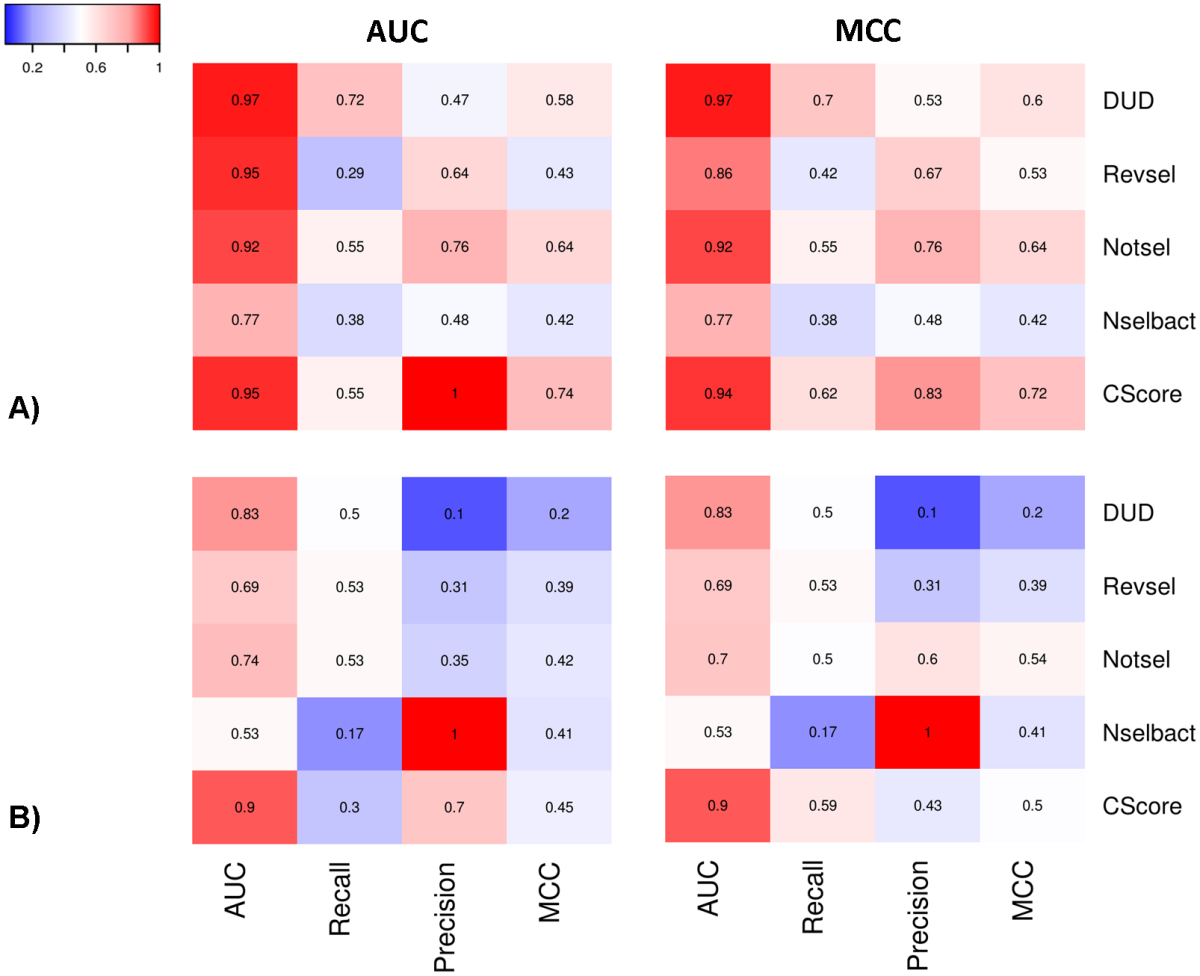
Heat maps comparing CScore with their component models for two strategies of selecting the best component models (highest AUC and MCC) and input data representations (A—for MACCS FP and a training ratio of 0.40; B—for SIFt-p generated using a 5-HT_1B_R template with loops and a training ratio of 0.40).

**Fig 7:**
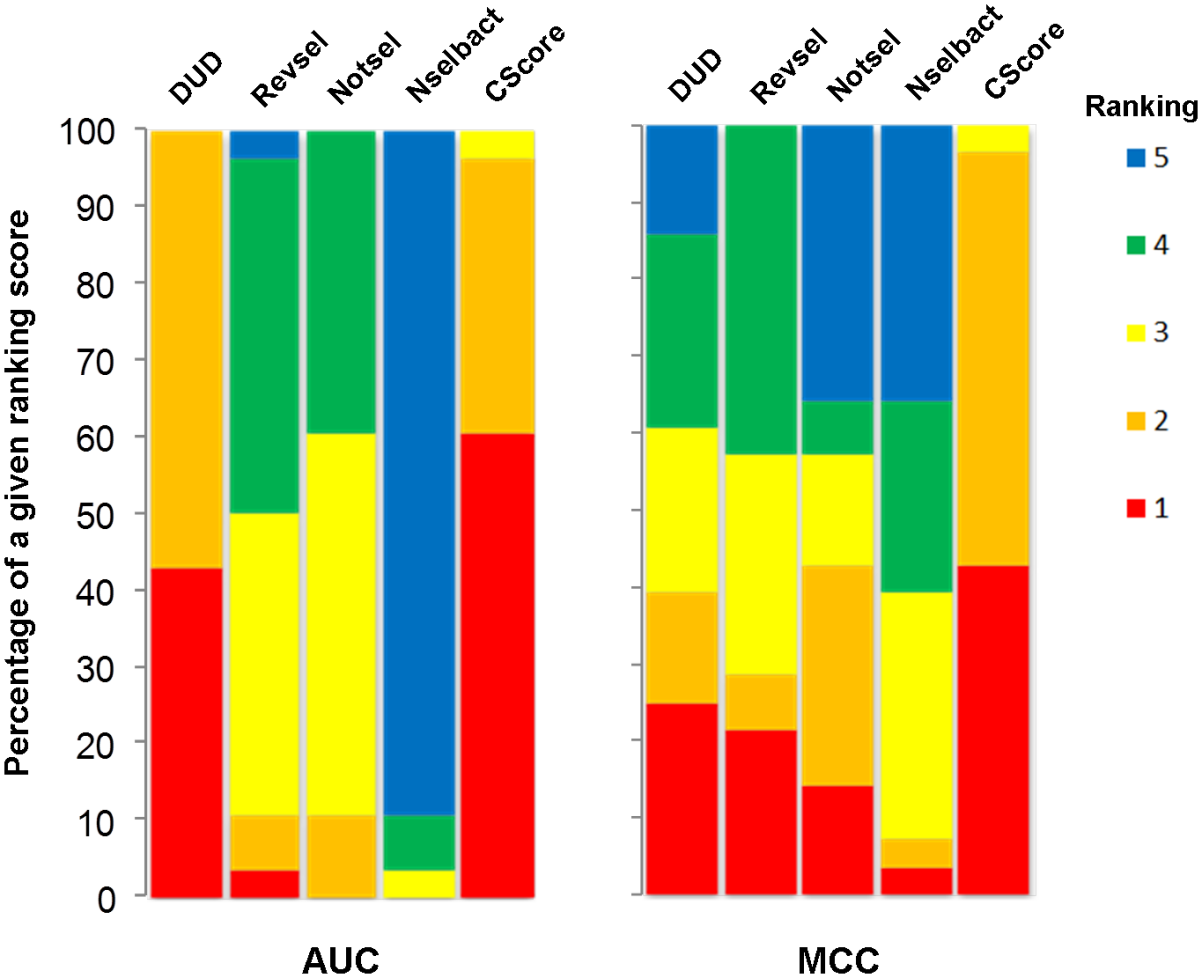
The ranking percentage (from 1 –best to 5 –worst) of a given SVM component and CScore model counted for AUC and MCC strategy.

To evaluate global performance, the CScore was compared with all component models. Fig 6 shows an example panel of heat maps comparing the results obtained for ligand-based (Fig 6A) and structure-based (Fig 6B) approaches. As expected, in the majority of cases, the CScore models were better than any of the component models. The CScore models optimized for AUC and MCC ranked first in 56.7% and 40% of the analyzed cases, respectively (Fig 7).

Additionally, to study the relationships between CScore and component models, the clustering of rows (represented by vectors containing four performance parameters) using the complete linkage method with Euclidean distance measure was performed (all of the mentioned heat maps are available in S1 File). The global analysis of dendrograms revealed that the performance of the CScore models was coupled with component models on different levels of performance similarity. For example, considering the highest level of similarity (i.e., the shortest Euclidean distance between two models), the performance of the CScore model was most similar to DUD, Revsel, Notsel and Nselbact in eight, six, eight and one cases, respectively. In the five remaining cases, CScore was coupled on the second level (i.e., with a cluster formed by two component models). Interestingly, eight cases had singleton component models (Nselbact and Notsel in six and two, respectively) and generally displayed poor performance.

**Fig 8:**
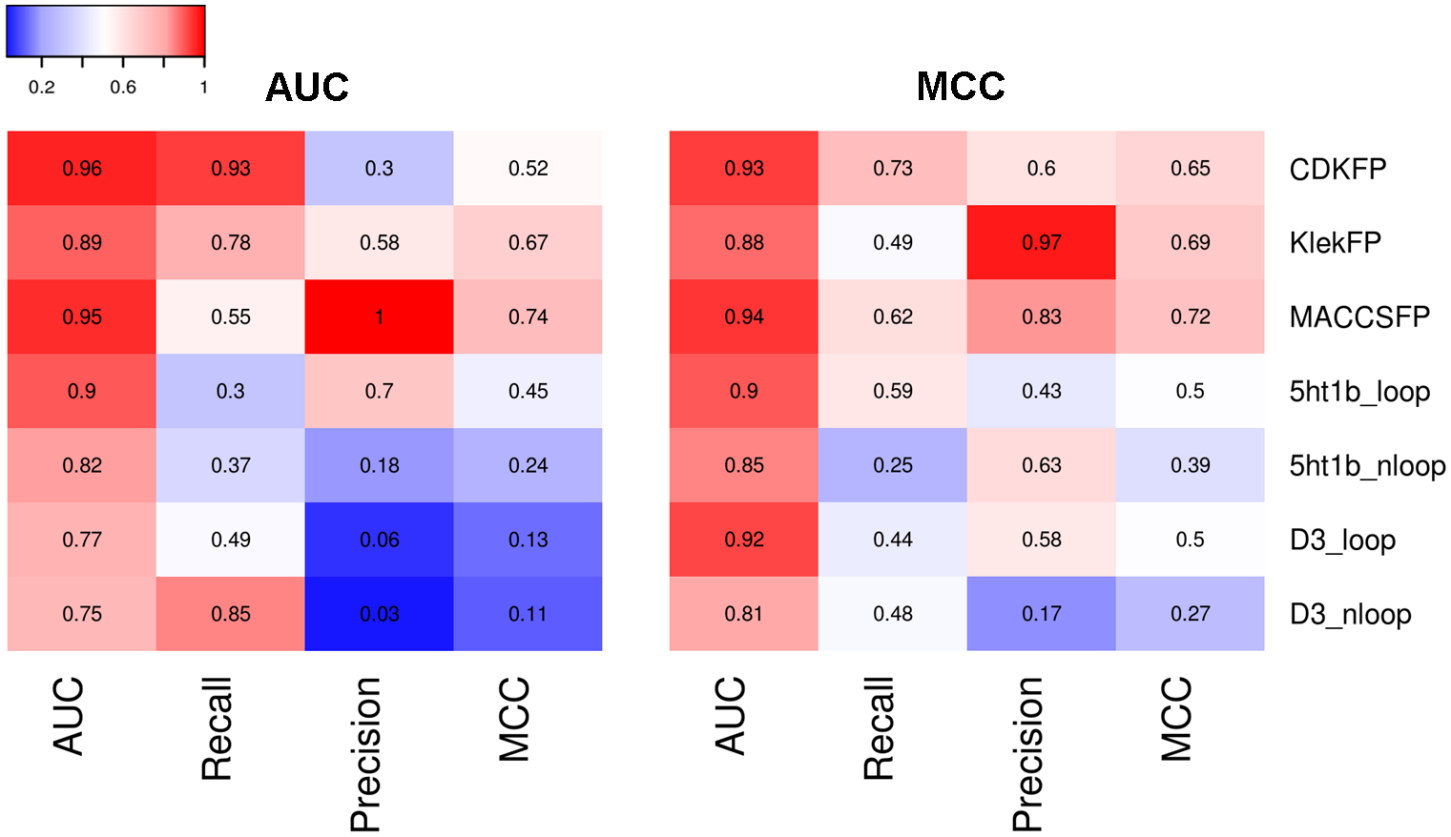
Heat maps comparing the performance of CScore models obtained for all the studied cases, i.e., representation of the data (three molecular fingerprints and SIFt-p for models with and without loops) and strategy for component models selection (AUC, MCC).

Comparison of the approaches used to generate the input data (Fig 8) revealed that significantly better CScore models were obtained for ligand-based (average BEDROC = 0.95, MCC = 0.67 and AUC = 0.96) compared with structure-based (average BEDROC = 0.79, MCC = 0.38, and AUC = 0.86) approaches. Among all the molecular fingerprints that were utilized, MACCS FP (BEDROC = 0.94, MCC = 0.70 and AUC = 0.95) and KlekFP (BEDROC = 0.95, MCC = 0.69 and AUC = 0.95) performed at a comparable level that was greater than that of CDKFP (average BEDROC = 0.94, MCC = 0.61 and AUC = 0.95).

Regarding the homology modeling approach, the results showed that for both 5-HT_1B_R and D_3_R templates, slightly better CScore models were obtained when homology models with extracellular loops were used for SIFt-p generation (MCC for both models = 0.50, Fig 8). Additionally, the template also appears to influence the performance of the CScore models—better results were obtained for more homologous 5-HT_1B_R template.

The CScore models obtained for component models selected using MCC criteria were slightly more efficient than those constructed using component models with the best AUC values. Because we optimized MCC to identify the threshold enabling the best classification effectiveness for each component model, the approach based on CScore model generation for MCC provides, globally, SVM models with the highest performance on classification tasks.

**Fig 9:**
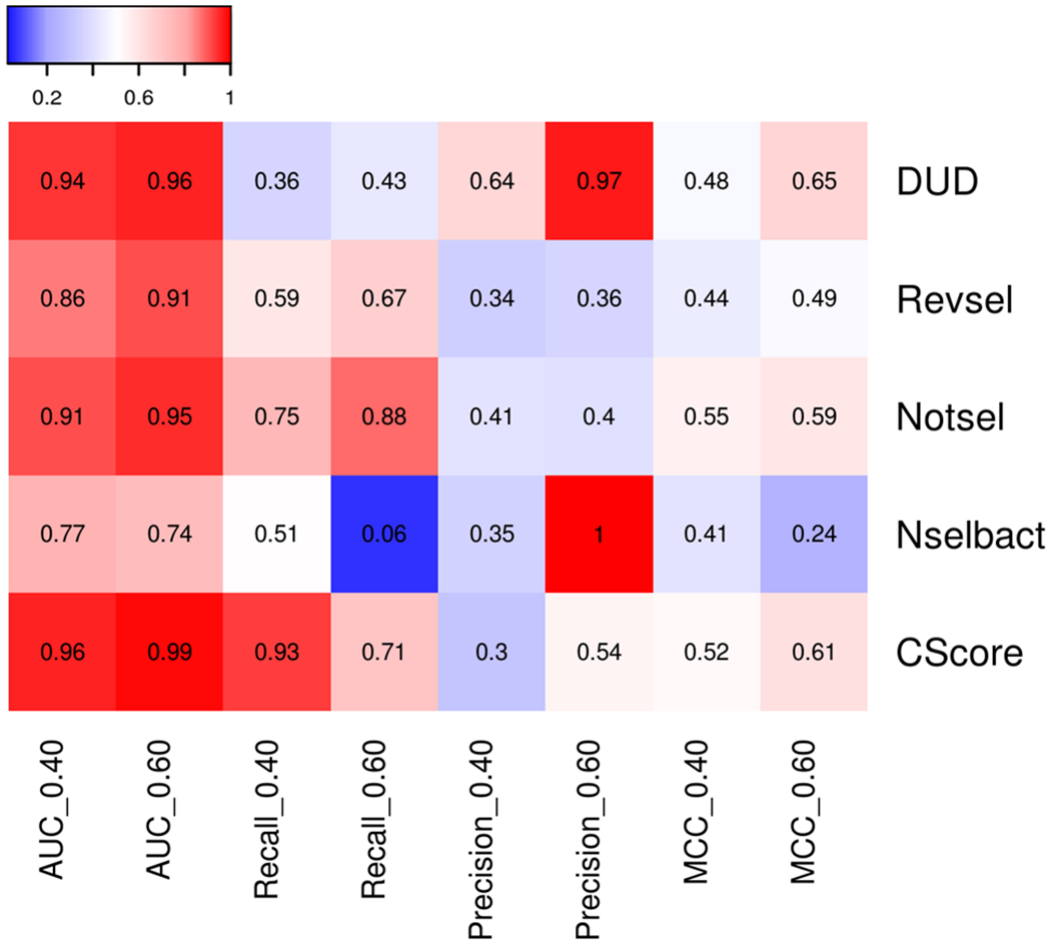
Comparison of the influence of training set size on the performance of separate models for MACCS FP.

Finally, increasing the size of the training set (from 40% to 60% actives) improved the performance of both component and CScore models in the majority of cases (Fig 9), which is consistent with our previous findings [62,63].

## Discussion

### Selectivity threshold

As demonstrated in the present analysis, machine learning classification models trained on a set of ligands with different selectivity and activity profiles can provide a consensus model, the performance of which is significantly better than the component models. It must be stressed that the ligand was regarded as selective for 5-HT_7_R as long as the ratio of *K*_i_(5-HT_1A_)/*K*_i_(5-HT_7_) was greater than 5. The rationale for this criterion was based on a close investigation of the data retrieved from ChEMBL database v17, which showed that there were 69 compounds for selectivity threshold ≥ 5, whereas there were only 34 compounds for threshold ≥ 20 that could be used for training and testing of the SVM models (S2 Fig). It should be noted that different thresholds for the selectivity index and rationale for their assessment have been used in similar studies, however, a quantitative definition is lacking. For example, Ma et al. used a selectivity index ≥ 10, which was selected based on the findings for the selective CB_1_/CB_2_ cannabinoid ligand by J.W. Huffman [10]. However, Wang et al. used a threshold for the selectivity index ≥ 3 for the kNN QSAR Classification model for 5-HT_1E_/5-HT_1F_ receptor selectivity [5]. To minimize errors resulting from, e.g., uncertainty regarding *K*_i_ values, they tested four selectivity thresholds and demonstrated that a threshold higher than 3 led to unacceptable models, generally due to a number of selective ligands that was too small. However, a higher selectivity index threshold was used by Wassermann [9] and Ning (50-fold) [8].

### Performance of the CScore

To test general performance, as well as the “early recognition” preferences of the proposed algorithm, different performance metrics were applied. Although widely used, AUC is not a sufficient metric to address the "early recognition" problem specific to VS [57,58]. Additionally, the application of AUC to rank any database necessitates the selection of a decision threshold that enables binary classification (e.g., active/inactive or selective/not-selective). Consequently, different metrics can potentially be used to identify the optimal threshold. For example, Alvarsson et al. used net reclassification improvement (NRI) [41]. Because MCC is a more balanced summary statistic of the confusion matrix when unbalanced classes (see Table 1) are used [57], we decided to apply it in our algorithm.

Our analyses revealed that significantly better CScore models were obtained when the component models with the best MCC compared with the best AUC value were selected. This observation is explained in Fig 5, in which red circles on ROC curves depict the decision thresholds that were determined by maximizing MCC. These circles are localized in the area of the curve in which the number of true positives is ranked before false positives, which in some cases corresponds to the “early recognition” of the BEDROC.

### Fingerprint influence

The influence of diverse parameters on the performance of the proposed algorithm was tested. Interestingly, CScore models based on the molecular fingerprints showed better performance than averaged interaction fingerprints (SIFt-p). All of the used fingerprints have different lengths, ranging from 166 (MACCS FP) to 4860 bits (Klekota-Roth FP), whereas concatenated SIFt-p had lengths of 1494 and 1458 bits for receptors with and without EL, respectively. There was no correlation between the length of the representation and the performance of the CScore model. The superior performance of ML models based on molecular rather than on interaction fingerprints in retrieving selectivity patterns may be due to uncertainty in predicting the correct binding mode by docking. It should be noted that because models obtained for receptors with EL showed better performance than those without loops, these additional four amino acids belonging to EL could play a role in the recognition of selective ligands.

The method presented herein could be especially useful for the virtual screening of chemical databases and for assessing combinatorial libraries to prioritize compounds for synthesis. It also offers more control capabilities in virtual screening searches for selective ligands because it enables the construction of a CScore model using different classification thresholds and performance parameters, e.g., one can generate a CScore model to optimize its performance for recall or precision. Additionally, the proposed algorithm is flexible, and after redefining the training classes, it can be used to, e.g., predict multimodal ligands.

## Conclusions

In this study, a new algorithm is presented to identify new target-selective ligands and is evaluated based on its selectivity prediction for 5-HT_7_ receptor ligands over the 5-HT_1A_ subtype. We adopted data fusion and SVM component models (class-specific) that were trained on four datasets, i.e., selective toward 5-HT_7_R (Selective) or 5-HT_1A_R (Revsel), not-selective (Notsel) and not-selective but active (Nselbact), to construct the consensus classifier—CScore. The primary objective of this study was to obtain a virtual screening algorithm, which was evaluated in terms of its “early recognition” performance using the BEDROC metric. The analyses showed that the CScore was a significantly better scoring strategy than the best single models trained in a classical manner and the best in-class component models. The selection of component models to construct the consensus classifier is crucial and is significantly influenced by the molecular representation and performance parameter applied. In all studied cases, selection of the component models with the best MCC versus AUC value improved “early recognition” (measured by BEDROC).

Considering the successful implementation of the proposed algorithm, it will be incorporated into our screening protocol [64] and applied to analyze combinatorial libraries to prioritize the synthesis of selective 5-HT_7_R ligands. Further improvements in the functionality of the algorithm will be conducted to improve its utility for other research groups (S2 File).

## Supporting Information

S1 Fig Heat map showing all average pairwise intra- and inter-class similarities calculated using the Tanimoto metric and CDK FP. (TIF)

S2 Fig Histogram of the compound selectivity index. (TIF)

S1 File Heat maps with row clustering comparing the CScore and component models developed for all ligand- and structure-based approaches to the data representation. (PDF)

S2 File A zip file containing scripts, datasets and optimized SVM models used in this study. (ZIP)
