# Supplementary figures and images for: An Algorithm to Identify Target-Selective Ligands – A Case Study of 5-HT7/5-HT1A Receptor Selectivity
Source: PLoS One. 2016 Jun 7;11(6):e0156986. doi: 10.1371/journal.pone.0156986 (PMC4896471; doi:10.1371/journal.pone.0156986)

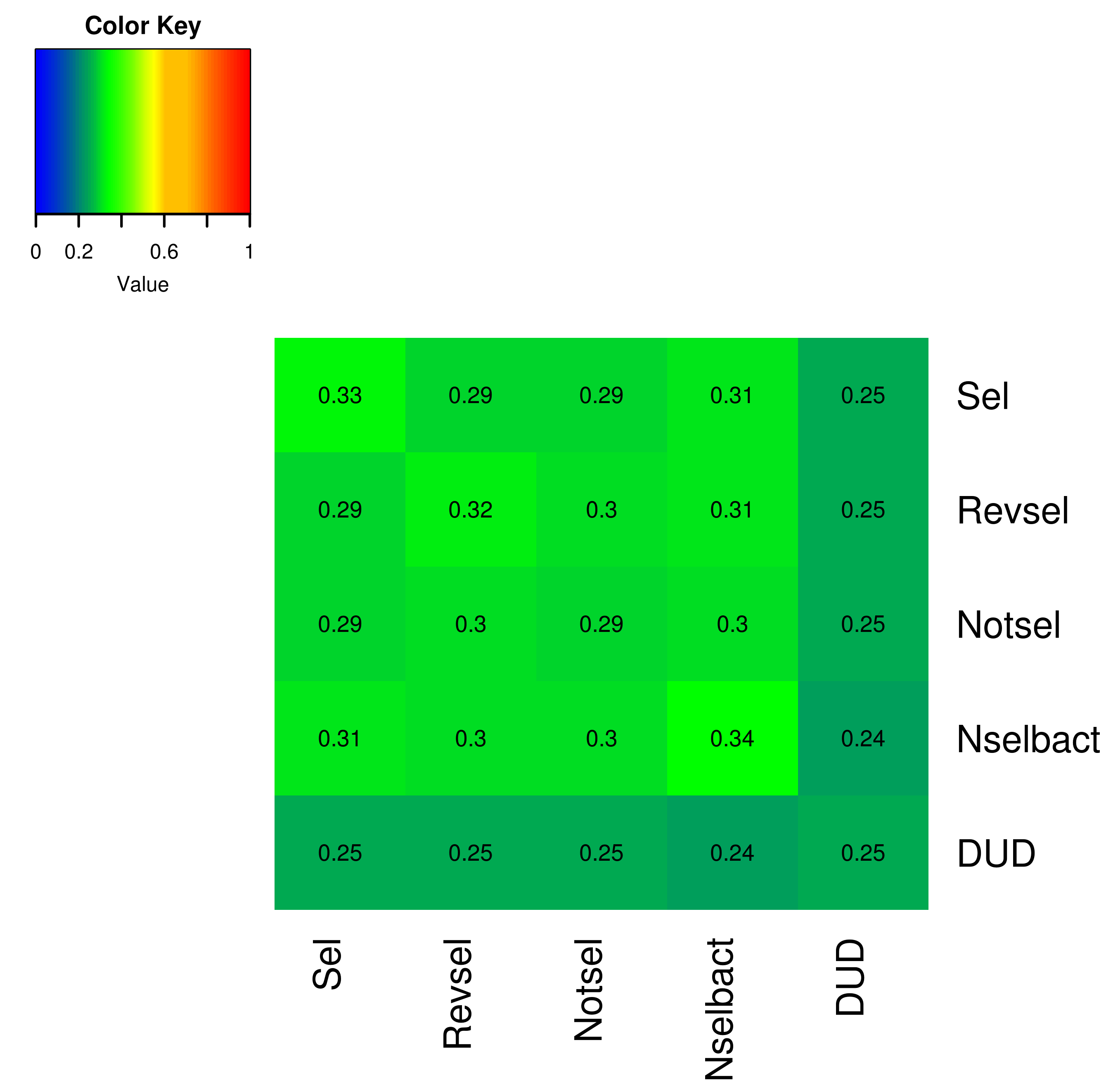

Supplement: S1 Fig — (TIF) [file pone.0156986.s001.tif]

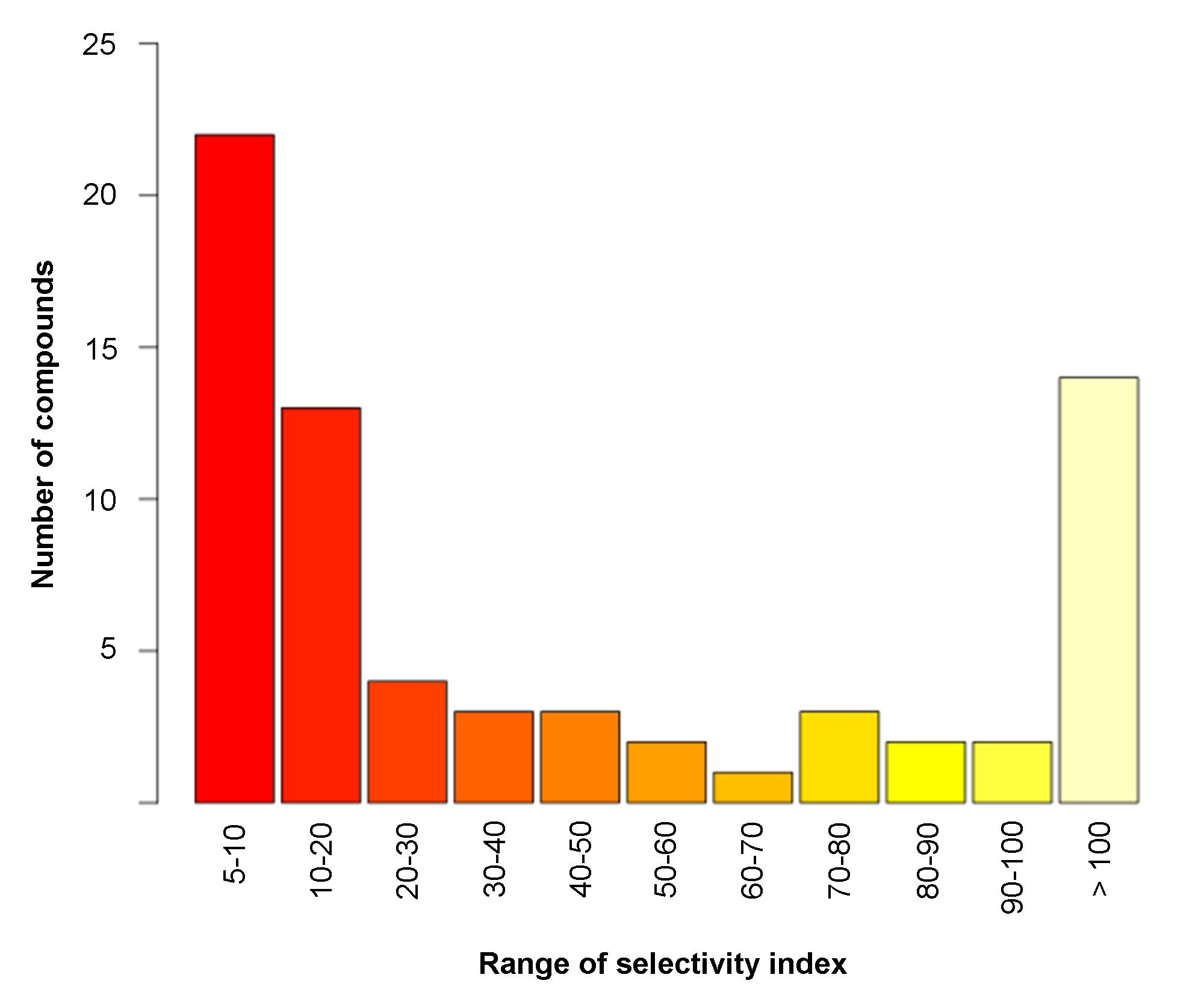

Supplement: S2 Fig — (TIF) [file pone.0156986.s002.tif]

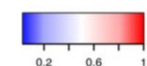

CDKFP

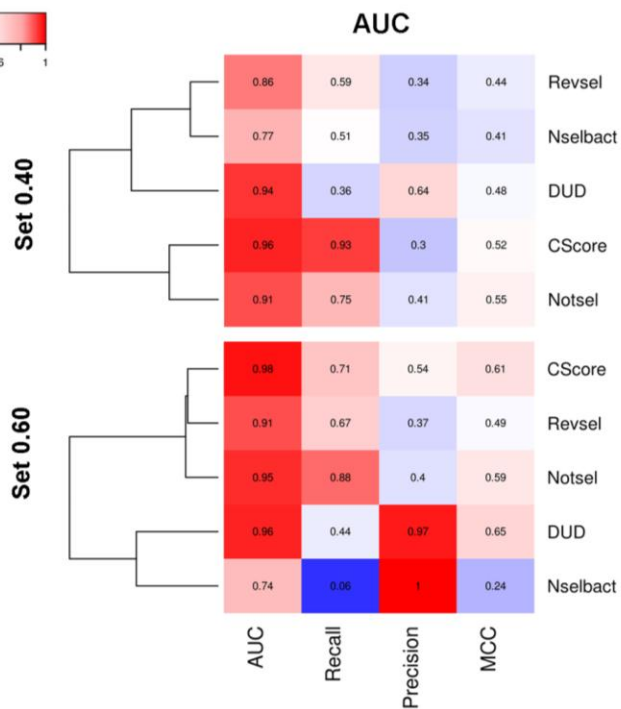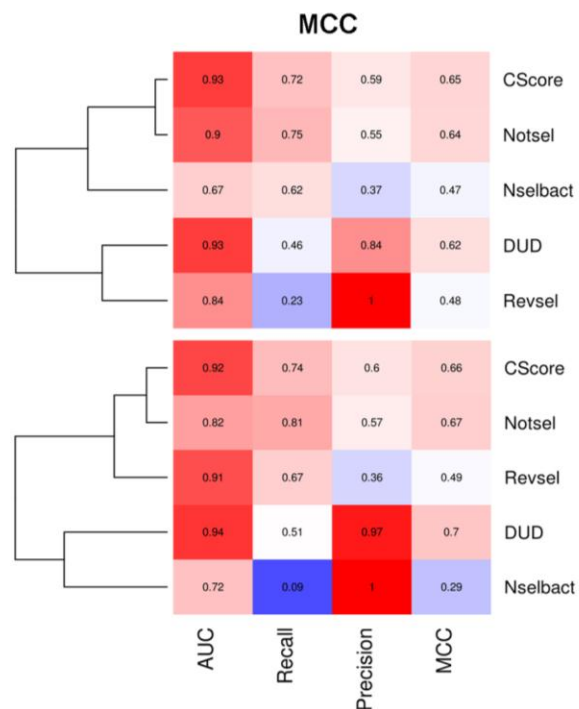

KlekFP

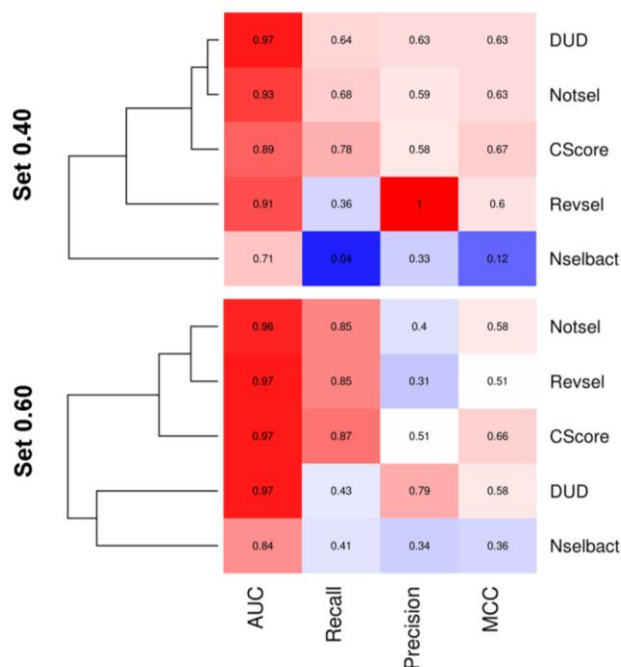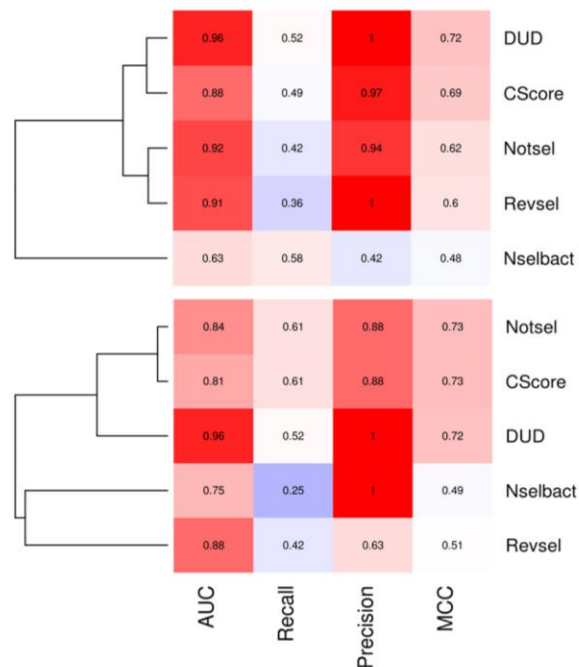

MACCSFP

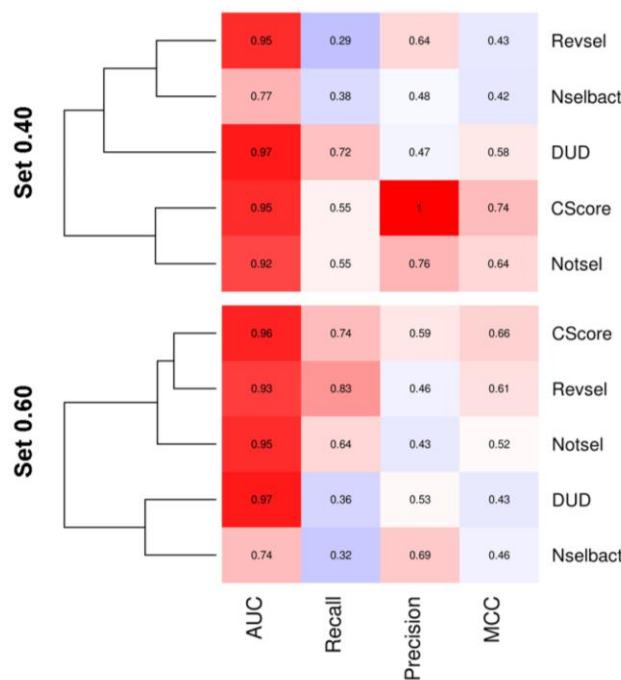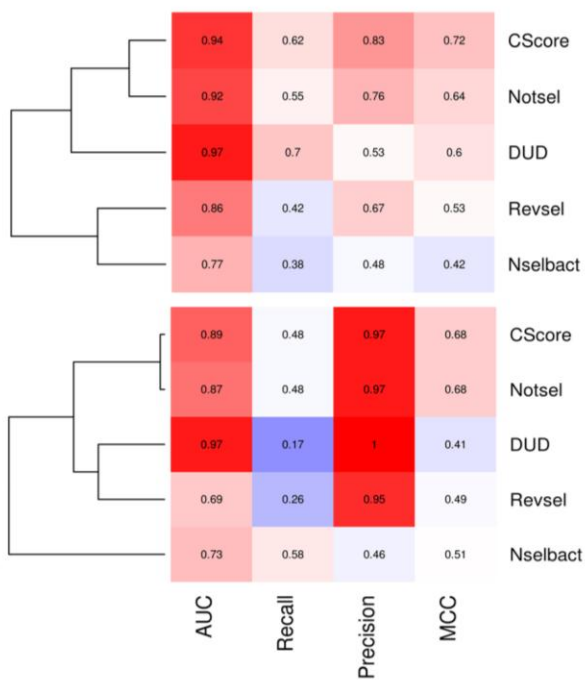

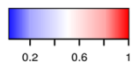

5-HT<sub>1B</sub> nloop

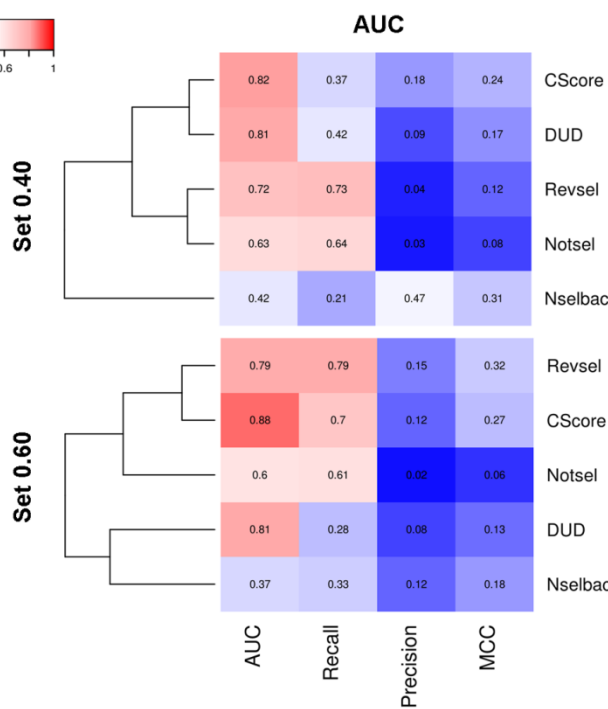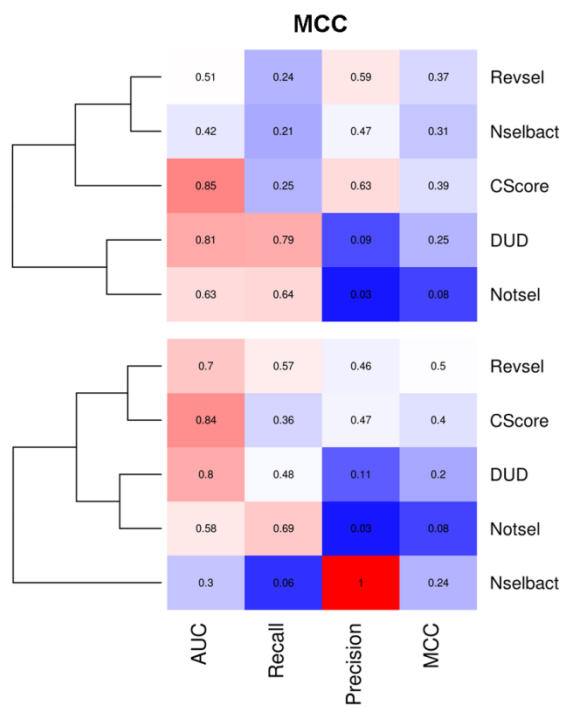

5-HT<sub>1B</sub> loop

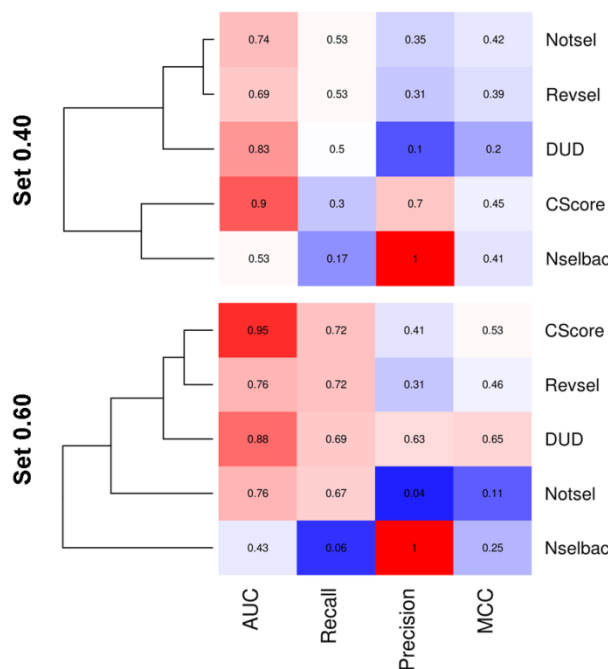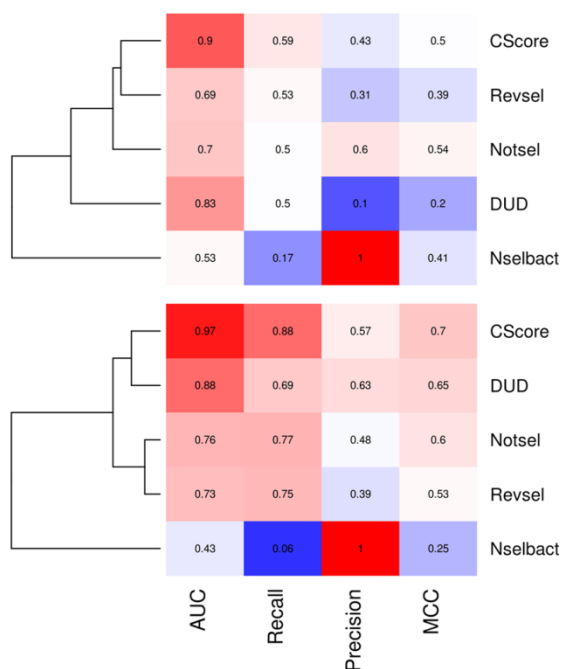

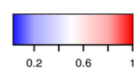

D<sub>3</sub> nloop

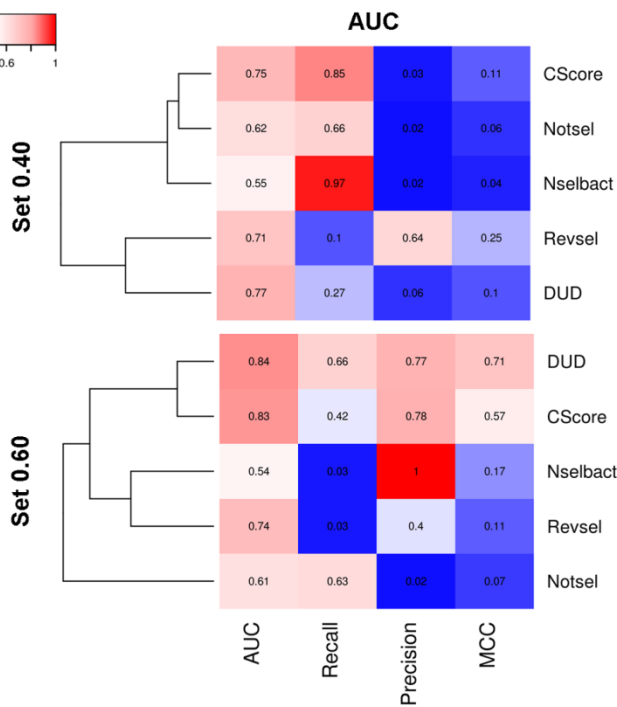

**MCC**

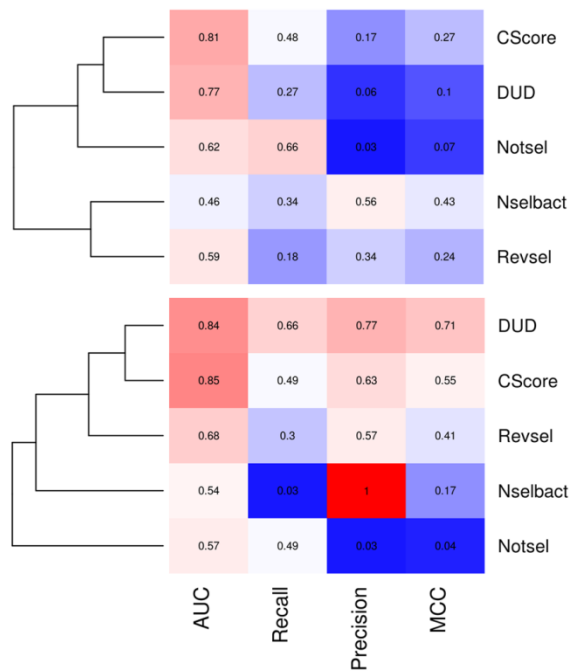

D<sub>3</sub> loop

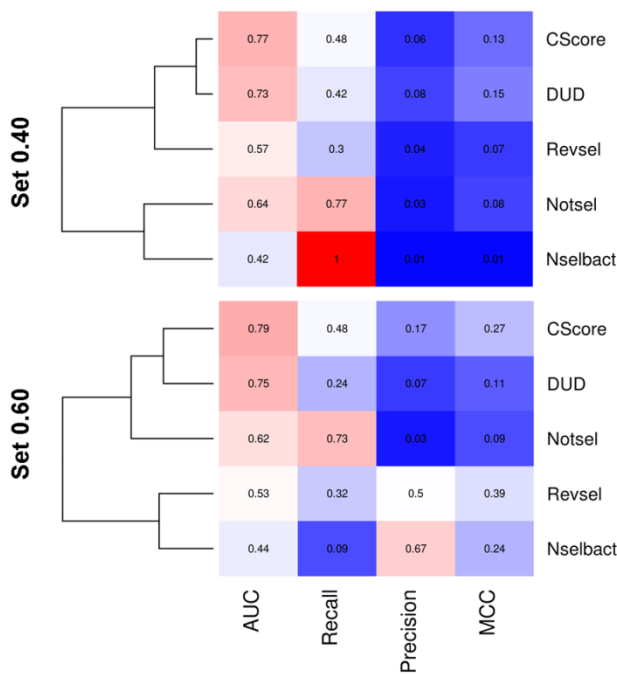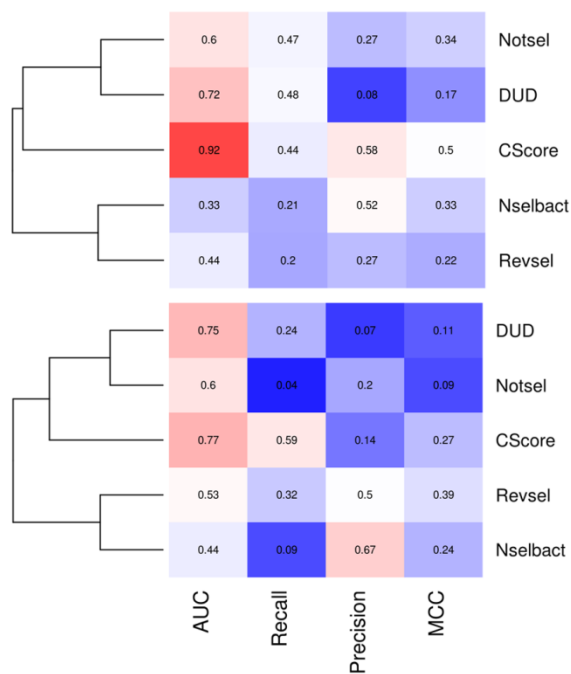

Supplement: S1 File — (PDF) [file pone.0156986.s003.pdf]
